# Comparison of Clinical and Laboratory Manifestations Between Acute Appendicitis and Mesenteric Lymphadenitis in Children

**DOI:** 10.7759/cureus.62437

**Published:** 2024-06-15

**Authors:** Ladan Shahba, Maryam Kuhestani Parizi, Mohammad Shafie

**Affiliations:** 1 Surgery Department, Kerman Medical University, Kerman, IRN

**Keywords:** appendectomy, laboratory manifestation, mesenteric lymphadenitis, children, appendicitis

## Abstract

Background and objectives: Acute appendicitis is a major cause of emergency surgery in children and adolescents. Mesenteric lymphadenitis is also one of the most common differential diagnoses in children with acute appendicitis, and despite its high prevalence, few clinical studies have examined its features. The clinical signs of mesenteric lymphadenitis in children are very similar to those of acute appendicitis. The aim of this study was to determine the clinical manifestations of acute appendicitis and mesenteric lymphadenitis in children.

Methods: In this cross-sectional study, patients less than 15 years old admitted to the pediatric emergency center of Afzalipour Hospital in Kerman from 2018 to 2021 were studied, and those who had a final diagnosis of appendicitis, mesenteric lymphadenitis, or appendectomy were included in the study by census. Data collection from the records of these patients included age, sex, clinical signs, duration of emergency until diagnosis, fever, number of times of vomiting, leukocytosis, lymphocyte, neutrophil count, neutrophil-to-lymphocyte ratio, sonographic findings, and pathology findings. SPSS software and descriptive and analytical statistics were used to analyze the data.

Results: The results showed that unlike sex (p=0.11), there was a significant difference between the two groups in terms of age (p<0.001). Nausea, vomiting, anorexia, pain shift, and leukocytosis were more common in the acute appendicitis group than in the mesenteric lymphadenitis group, with a significant difference between the two groups (p<0.001). There was a significant difference between the two groups in terms of neutrophil percentage and neutrophil-to-lymphocyte ratio (p<0.001).

Conclusion: The present study was conducted with the aim of assisting medical professionals due to the possibility of misdiagnosing mesenteric lymphadenitis with acute appendicitis. Differentiation of these two diseases is facilitated by the significant differences in their clinical signs and test results. These results can be a useful guide for physicians to better diagnose the diseases.

## Introduction

Acute appendicitis (AA) is the inflammation of the appendix because of the obstruction of its lumen by fecal matter, parasites, and food [1,2]. AA is the greatest usual indication for emergency surgery, occurring more often in children and young adults than in patients of advanced age [3]. Also, it ranks as the most frequent reason for abdominal surgery in children, with an annual diagnosis rate of 60,000-80,000 cases in North America [4]. The presentation of AA can be influenced by its location, but vomiting, diarrhea, fever, and right groin pain with tenderness on examination are the commonly found presentations in patients with AA [5]. Laboratory findings, such as white blood cell count, the level of the C-reactive protein, and sonography are useful tools for diagnosing AA [6]. Needless to say, the diagnosis of AA in children in time plays an important role in preventing its complications and mortality [2].

Mesenteric lymphadenitis (ML) is caused by the inflammation of the lymph nodes of the membrane connecting the abdominal wall to the intestines. In other words, in ML, the size of the mesenteric lymph nodes increases. This self-limited disease is recognized as among the prevalent alternative diagnoses in children who are suspected of having AA [7,8]. ML or mesenteric adenitis is caused by inflammatory disorders, infections, mostly viral pathogens, or malignancy and is usually defined as a collection of three or more lymph nodes with a short axis diameter greater than 5 millimeters in the CT scan [9,10]. ML presentation is very similar to that of AA [11]. Abdominal pain, fever, diarrhea, nausea, and vomiting are the usual signs and symptoms of ML, which can cause acute or chronic inflammation, depending on the causative microorganism [12]. The abdominal pain of ML is mostly located in the right lower quadrant, but it can be more widespread [13]. Studies have shown that accurate differentiation of ML from AA in children is not possible only by examination and sonography is necessary in inconclusive cases [11]. It should be noted that distinguishing between ML and AA is challenging even for experienced physicians [7].

As mentioned before, although ML, which has a high prevalence, is one of the most frequent differential diagnoses in children with AA, few clinical studies have investigated the characteristics of ML. Moreover, considering the similar symptoms and different treatments of these two diseases, it is very important to distinguish them from each other. Therefore, this study was undertaken in Kerman with the purpose of comparing the clinical and laboratory signs and symptoms of the two diseases.

## Materials and methods

Research setting, population, and the sample size

We performed this cross-sectional research in the emergency room (ER) of Afzalipour Hospital in Kerman. Afzalipour Hospital, which is one of the largest hospitals in Iran with one of the busiest emergency departments, was established in 2004 [14]. We included 370 children with an early diagnosis of AA or ML from 2018 to 2021, by census. Two hundred and seventy-four of these children were diagnosed with AA and 70 with ML, and 26 were excluded from the study due to lack of access to their files.

Inclusion and exclusion criteria and ethical considerations

The study's inclusion criteria include age less than 15 years, referring to the ER of Afzalipour Hospital, and final diagnosis with appendicitis or mesenteric lymphadenitis or the appendectomy treatment due to AA. Also, children who were discharged with a diagnosis different from their primary one and patients who left the hospital on their own will before the final diagnosis, discontinued their treatment, or received appendectomy treatment for reasons other than AA were excluded from the study. The data of the cases were collected after approval by the university, anonymously and without checking the patients' personal information in the information form. The Ethics Committee of the Kerman Medical University reviewed and approved this study, and its ethics code is IR.KMU.AH.REC.1398.126.

The method and tools of data collection

The data collection tool used in the current study was a researcher-made checklist, which included two parts: the patient's demographic information and disease-related variables. The collected variables included age, sex, patient's clinical signs and symptoms, such as fever, nausea, and vomiting, the duration of the patient's stay in the ER until their diagnosis, laboratory findings, sonography reports, and pathology reports. The laboratory findings included leukocytosis rate, lymphocyte count, neutrophil count, and neutrophil-to-lymphocyte ratio. After coordinating with the associated officials at Kerman Medical University and Afzalipour Emergency Department, the researcher collected the required data and entered them into anonymous and coded forms.

Data analysis tools and methods

We conducted an analysis of the collected data utilizing descriptive statistics, chi-squared test, and independent t-test. Also, we performed all analyses using IBM SPSS Statistics for Windows, Version 29.0 (Released 2023; IBM Corp., Armonk, New York, United States) with a significance level of p<0.05.

## Results

The entire number of samples was 370, none of whom had any immunodeficiency disorders. Out of these, 274 patients were diagnosed with AA, and 70 with ML. Twenty-six of the cases were excluded due to the lack of access to the required information. The results showed the most important for surgery in these patients were inflamed and purulent appendix, 85.8% and 84.2%, respectively.

Patients had an average age of 10.4±3.34 and 6.77±2.65 years for AA and ML, respectively. Statistical significance was observed in this disparity (p<0.001). The majority of the patients in both AA and ML groups were males, 53.4% and 64.3%, respectively, but there was no statistically significant divergence in the sex of the patients (p=0.11). Also, the use of antibiotics before the surgery in AA patients was 4.2%, which was less than their use in ML patients, with 13.4%. The observed dissimilarity was significant (p=0.009) (Table 1).

**Table 1:** The examination of the demographic variables and the antibiotic consumption status in the patients of the current study *chi-squared test; **Mann-Whitney U test

Variable		Acute appendicitis	Mesenteric lymphadenitis	Effect size (p-value)
Age (years)		10.4±3.34	6.77±2.65	0.412 (<0.001**)
Sex	Male	147 (53.6%)	45 (64.3%)	0.079 (0.11*)
	Female	127 (46.4%)	25 (35.7%)	
Antibiotic consumption	No	250 (95.8%)	58 (86.6%)	0.139 (0.009*)
	Yes	11 (4.2%)	9 (13.4%)	

Most patients with AA had leukocytosis (39.1%), and those with ML had normal white blood cell counts (62.5%), which demonstrated a significant statistical difference (p<0.001). The positive predictive value of severe leukocytosis, i.e., white blood cell count of more than 15000, in AA was calculated at 91%. In other words, the probability of having AA in patients who had severe leukocytosis was 91%.

The number of neutrophils in 52% of AA cases was between 70% and 84%, and it was less than 70% in 71.2% of ML cases, with a noticeable difference between AA and ML groups (p<0.001). Furthermore, the average number of lymphocytes was 17.58+13.42 and 34.95+17.64 in AA and ML cases, respectively, which was a considerable difference (p<0.001) (Table 2).

**Table 2:** Blood factors and duration of the onset of symptoms and hospitalization in the current study's participants *chi-squared test; **Fisher's exact test; ***Mann-Whitney U test

Variable		Acute appendicitis	Mesenteric lymphadenitis	Effect size (p-value)
White blood cells	Less than 10,000 (normal)	68 (25.6%)	40 (62.5%)	0.300 (<0.001*)
	10,000-15,000 (mild leukocytosis)	104 (39.1%)	15 (23.4%)	
	More than 15,000 (severe leukocytosis)	94 (35.3%)	9 (14.1%)	
Neutrophils	Less than 70%	55 (24.2%)	42 (71.2%)	0.391 (<0.001*)
	70-84%	118 (52.0%)	13 (22.0%)	
	More than 84%	54 (23.8%)	4 (6.8%)	
Neutrophil-to-lymphocyte ratio	Less than 3.5	59 (26.0%)	39 (68.4%)	0.348 (<0.001*)
	3.5 or higher	168 (74.0%)	18 (31.6%)	
Platelets	Less than 150	7 (2.7%)	3 (5.2%)	0.054 (0.57**)
	150-450	232 (89.6%)	50 (86.2%)	
	More than 450	20 (7.7%)	5 (8.6%)	
The duration of the symptoms before visiting the ER in hours		53.7+73.29	77.32+86.43	0.165 (0.002***)
The duration of hospitalization in the ER until the diagnosis confirmation in hours		8.13+24.38	21.20+07.51	0.367 (<0.001***)

The neutrophil-to-lymphocyte ratio was 3.5 or higher in 74% of patients with AA and was less than 3.5 in 68.4% of patients with ML, with a significant difference (p<0.001). The positive predictive value of the neutrophil-to-lymphocyte ratio in the AA cases was calculated to be 91%, i.e., the probability of having AA was 91% in patients with a neutrophil-to-lymphocyte ratio of 3.5 or higher.

The duration of the onset of symptoms until referring to the hospital in hours was 53.7+73.29 in AA and 77.32+86.43 in ML patients, which showed a significant difference between the two groups of patients (p=0.002). The duration of hospitalization in the ER until confirmation of the diagnosis in hours was 8.13+24.38 and 21.20+07.51 hours in AA and ML patients, respectively, which signified a notable divergence (p<0.001) (Table 2).

The most common symptoms in AA were nausea (89.4%), loss of appetite (86%), and vomiting (81.8%) in AA patients and vomiting (58%), nausea (53.6%), and fever (51.4%) in ML patients, with AA cases experiencing more nausea, vomiting, loss of appetite, and pain shifting than patients with ML with a statistically notable contrast between these groups (p<0.001). Also, the positive predictive value of pain shifting in AA patients was 65%, i.e., 65% of patients with pain shifting had AA.

**Figure 1:**
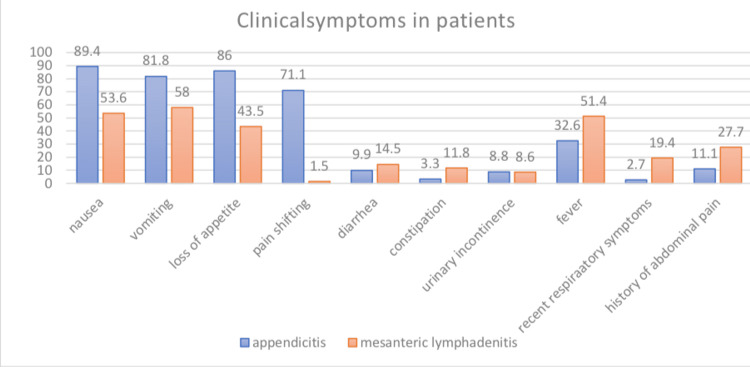
The frequency distribution of clinical symptoms in patients with AA and ML AA: acute appendicitis; ML: mesenteric lymphadenitis

Fever (p=0.005), history of recent respiratory symptoms (p<0.001), and abdominal pain (p=0.001) were considerably more in ML compared to AA cases. The positive predictive value of history of recent respiratory symptoms was 91% in ML cases, i.e., the probability of having ML in people with a recent history of respiratory symptoms was 91% (Figure 1).

Tenderness was more common in the AA patients compared to the ML patients, which shows a significant difference in tenderness (p<0.001). Tenderness in AA was located more frequently in the lower right quadrant, which was significantly different from ML (p<0.001), with tenderness mostly reported in the periumbilical region. While 37.9% of AA patients experienced rebound tenderness, there was no rebound tenderness in ML patients. These two groups showed a statistically significant variation in this regard (p<0.001) (Table 3).

**Table 3:** Examination results in the current study's participants *chi-squared test; **Fisher's exact test LLQ: left lower quadrant; RLQ: right lower quadrant; LUQ: left upper quadrant; RUQ: right upper quadrant

Examination			Acute appendicitis	Mesenteric lymphadenitis	Effect size (p-value)
Tenderness		No	10 (3.7%)	32 (47.1%)	0.514 (<0.001*)
		Yes	263 (96.3%)	36 (52.9%)	
Tenderness location	Generalized	No	248 (94.3%)	30 (83.3%)	0.111 (0.02**)
		Yes	15 (5.7%)	6 (16.7%)	
	LLQ	No	247 (93.9%)	34 (94.4%)	0 (>0.99**)
		Yes	16 (6.1%)	2 (5.6%)	
	RLQ	No	32 (12.2%)	31 (86.1%)	0.577 (<0.001*)
		Yes	231 (87.8%)	5 (13.9%)	
	Periumbilical area	No	236 (89.7%)	17 (47.2%)	0.369 (<0.001*)
		Yes	27 (10.3%)	19 (52.8%)	
	Hypogastric area	No	257 (97.7%)	33 (91.7%)	0.085 (0.08**)
		Yes	6 (2.3%)	3 (8.3%)	
	Suprapubic area	No	259 (98.5%)	36 (100.0%)	0 (>0.99**)
		Yes	4 (1.5%)	0 (0.0%)	
	RUQ	No	258 (98.1%)	35 (97.2%)	0 (>0.99**)
		Yes	5 (1.9%)	1 (2.8%)	
	Epigastric area	No	259 (98.5%)	33 (91.7%)	0.112 (0.04**)
		Yes	4 (1.5%)	3 (8.3%)	
	LUQ	No	263 (100.0%)	36 (100.0%)	-
		Yes	-	-	
Rebound tenderness		No	169 (62.1%)	68 (100.0%)	0.321 (<0.001*)
		Yes	103 (37.9%)	0 (0.0%)	
Percussion tenderness		No	1 (0.9%)	0 (0.0%)	0 (>0.99**)
		Yes	111 (99.1%)	14 (100.0%)	
Rovsing's sign		No	9 (8.0%)	1 (7.1%)	0 (>0.99**)
		Yes	103 (92.0%)	13 (92.9%)	
Cough sign		No	102 (91.1%)	14 (100.0%)	0.057 (0.25**)
		Yes	10 (8.9%)	0 (0.0%)	
Body temperature		<37.5	188 (70.7%)	42 (64.6%)	0.097 (0.36**)
		37.5-38.5	62 (23.3%)	15 (23.1%)	
		38.5-39	14 (5.3%)	7 (10.8%)	
		>39	2 (0.8%)	1 (1.5%)	

Turning to the imaging, while all of the ML patients had sonography, 12 of the suspected AA patients did not have sonography. The positive predictive value of sonography in the AA group was 78%, which displayed that the possibility of having AA in people who were positive for evidence in sonography was 78%. A limited number of patients had CT scans (seven patients in the AA and one in the ML group). Moreover, 24 AA and four ML patients had an X-ray.

**Figure 2:**
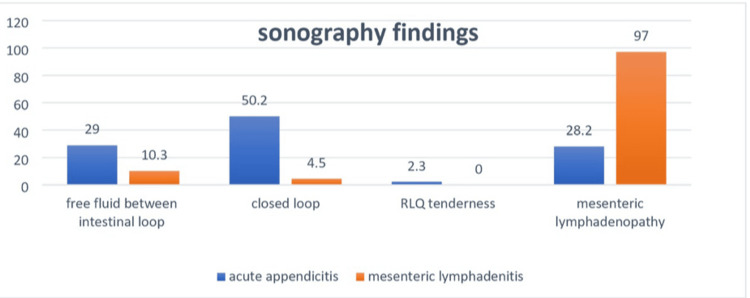
The frequency of sonography findings in the current study's participants

Of the cases, 29% and 10.3% (p=0.004) were positive for free fluid between the intestinal loop, and 50.2% and 4.5% (p<0.001) were positive for closed loop in AA and ML patients, respectively, which was significantly different for both variables. Mesenteric lymphadenopathy, i.e., 10 mm or higher lymph node axis diameter in sonography, was reported in 28.2% of AA and 4.5% of ML cases [15]; a statistically meaningful distinction was observed between these two groups (p<0.001) (Figure 2).

## Discussion

The outcome of this study indicated that while the most common symptoms in AA patients were nausea, loss of appetite, and vomiting, in ML, patients mostly experienced vomiting, nausea, and fever. Also, the results of Ghasemi et al.'s study in 2015 in Zabol demonstrated that there was a significant relation between AA and loss of appetite, which is consistent with the present study [16]. In Humes and Simpson's study in 2006 in England, abdominal pain was the most common presenting complaint of patients with ML [5]. This difference may be due to the use of painkillers or homemade herbal medicine to treat the abdominal pain and discomfort of the patient before going to a medical center. In Gross et al.'s study in 2017, pain migration, vomiting, and abdominal pain were significantly more common in children with AA compared to those with ML [7]. In the current study, in addition to the symptoms mentioned by Gross et al. [7], vomiting and loss of appetite were also more common symptoms in AA patients compared to ML patients. Moreover, in our study, the positive predictive value of pain shift in the AA group was 65%. Also, the results of Chanchlani's research on children with ML in 2015 in India illustrated that the common clinical symptoms of this disease are fever, diarrhea, nausea, and vomiting [12]. These results were in line with the outcomes of our study. 

In our research, the time between the beginning of symptoms and going to the ER was significantly longer in the ML than in the AA group. In Gross et al.'s study, ML patients took longer after symptom onset to go to the ED [7], which is the same as the results of our research. Additionally, in physical examinations, the frequency of tenderness was higher than its absence, which is consistent with the results of Syed and Naji's study in 2021 in Dubi [17]. Besides that, in the results obtained from Ghasemi et al.'s paper, a significant statistical connection was observed between AA and rebound tenderness [16]. These results are largely consistent with the current study.

Laboratory results showed that most patients with AA had leukocytosis, high white blood cell and neutrophil levels, and neutrophil-to-lymphocyte ratios (3.5 or higher) compared to patients with ML. On the contrary, the average number of lymphocytes in AA patients was lower than in ML patients. Gross et al. found that while the ML group had higher lymphocyte counts than the AA group, the white blood cell levels and neutrophil-to-lymphocyte ratio were higher in patients with AA [7]. Moreover, in a systematic review and meta-analysis of more than 8000 patients with AA in 2020, Hajibandeh et al. showed that a neutrophil-to-lymphocyte ratio of 4.7 or higher predicts both the diagnosis and the severity of AA [18]. These findings support the observations of this study. Moreover, the outcome of Asadi Amoli et al.'s study in 2023 in Babol displayed that neutrophil counts and leukocytosis were significantly higher in AA patients than ML patients [19]. 

The positive predictive value of sonography in the AA group in the present research was 78%, which indicates a good value. This means the probability of AA in patients positive of evidence in sonography was 78%. The results of Toorenvliet et al. in 2011 in the Netherlands showed a positive predictive value of 96% for sonography, and the writers reported that differentiating AA from ML in children is not possible by using only clinical evaluation and sonography is necessary for a definitive diagnosis [11]. Sonography is the best tool for a quick distinction between AA and ML. Needless to say, with an accurate diagnosis, surgical intervention can be avoided because most cases of ML resolve with conservative treatment [12].

The cross-sectional nature was among the limitations of the current study, and the lack of access to patients resulted in the loss of parts of data, including CT scans and abdominal imaging findings. Besides that, it is advisable to proceed with caution in generalizing the findings of this study. This is because this study was limited to one treatment center. Although many patients visit the ER of Afzalipour Hospital, it is suggested that analytical studies on larger populations be conducted in other centers.

The results of this study pertain to patients with normal immune system status. A new study should be designed for immunocompromised individuals, considering the impact on the progression of symptoms and clinical laboratory results.

## Conclusions

In this study, the symptoms of AA patients were nausea, loss of appetite, and vomiting, and the symptoms of ML patients were vomiting, nausea, and fever, in order of frequency. Regarding the laboratory results, most of the patients with AA had higher white blood cell and neutrophil counts compared to patients with ML. Also, in AA patients, the neutrophil-to-lymphocyte ratio was 3.5 or higher, which was higher than that of ML patients.

Besides that, patients with ML go to the ER later than patients with AA. Due to the passage of time from the onset of symptoms, ML patients are diagnosed later than AA patients. ML patients also have more vague symptoms, which can make the diagnosis more challenging for a physician.

According to the findings from the current research, although a notable contrast was seen in signs, symptoms, clinical examinations, and laboratory findings between AA and ML, which can be helpful in the diagnosis, due to inconsistencies with the findings of other similar studies, a set of symptoms should be considered for correct diagnosis rather than just one sign.
